# Postoperation of cervical cancer with intestine metastasis: a case report and literature review

**DOI:** 10.1186/s12957-015-0759-3

**Published:** 2016-01-06

**Authors:** Xiuyan Yu, Zhen Wang, Zhigang Zhang, Yang Liu, Jian Huang

**Affiliations:** 1 Cancer Institute (Key Laboratory of Cancer Prevention and Intervention, China National Ministry of Education, Key Laboratory of Molecular Biology in Medical Sciences, Zhejiang Province, China), The Second Affiliated Hospital, Zhejiang University School of Medicine, 88 Jiefang Road, Hangzhou, Zhejiang Province 310009 China; 2 Department of Surgical Oncology, The Second Affiliated Hospital, Zhejiang University School of Medicine, 88 Jiefang Road, Hangzhou, Zhejiang Province 310009 China; 3 Department of Gynecology, The Second Affiliated Hospital, Zhejiang University School of Medicine, 88 Jiefang Road, Hangzhou, Zhejiang Province 310009 China

**Keywords:** Intestinal obstruction, Cervical cancer, Metastasis, Ileum, Sigmoid colon

## Abstract

**Background:**

Cervical cancer can infiltrate locally and directly spread to adjacent organs including the vagina, peritoneum, urinary bladder, ureters, rectum, and paracervical tissue, but the intestine metastasis from cervical cancer is extremely rare, which can easily be misdiagnosed.

**Case presentation:**

Here, we report a case about a 45-year-old postoperative cervical cancer patient with metastases to small intestine and sigmoid colon who presented abdominal distention and dull pain due to intestinal obstruction. The patient underwent exploratory laparotomy, and two intestinal segments including the tumors were resected. The postoperative pathological diagnosis illustrated sigmoid colon and terminal ileum metastatic squamous cell carcinoma.

**Conclusions:**

This case demonstrates that intestine metastasis must be considered in the differential diagnosis of acute abdomen in patients with cervical cancer even at an early tumor stage.

## Background

Cervical cancer is a worldwide disease which ranks as the second most common malignant disease and also the third most common cause of cancer death among women [1]. More than 90 % of cases are attributed to human papillomavirus (HPV) infection [2]. Most cases occur in developing countries, as no effective screening procedures are available [3]. Over the past decades, the survival of patients with cervical carcinoma has significantly improved attributing to early screening and the rapid development of concurrent cisplatin-based chemotherapy and radiotherapy [4, 5]. In spite of the prolonged survival, the patients are also at an increased risk of recurrence and metastases, which are the main causes of death. The primary routes in cervical carcinoma metastases are direct local extension and lymphatic dissemination, while hematogenous dissemination occurs infrequently, which usually occurs with advanced tumor or uncommon pathologic types, such as adenosquamous or neuroendocrine tumors. In general, cervical cancer can spread to adjacent organs including the vagina, peritoneum, urinary bladder, ureters, rectum, and paracervical tissue. Meanwhile, Common distant metastatic sites include the lungs, bones, and liver [6], but cervical cancer metastases to the small intestine and sigmoid colon are rare. Here, we present the first reported case of a cervical cancer patient with simultaneous metastases to the small intestine and sigmoid colon, based on our knowledge.

## Case presentation

A 45-year-old woman was admitted to our department with a 5-day history of abdominal distention and dull pain, especially at the upper umbilical region, and vomiting of gastric content without passage of stools or flatus. She had an erect abdominal plain radiograph which showed intestinal obstruction in a local hospital previously.

A careful medical history was taken on admission. Three years ago, she suffered from cervical cancer and had a radical hysterectomy with adnexectomy. The postoperative pathological diagnosis is cervical moderately differentiated squamous cell carcinoma with its maximum diameter to 9 mm and infiltration depth to 2 mm. No lymph node metastasis was found in the bilateral pelvic and common iliac lymph node. It was diagnosed with International Federation of Gynecology and Obstetrics (FIGO) stage IB1 cervical cancer. After the operation, she had a 3- to 6-month follow-up but did not have any radiotherapy or chemotherapy.

**Fig. 1:**
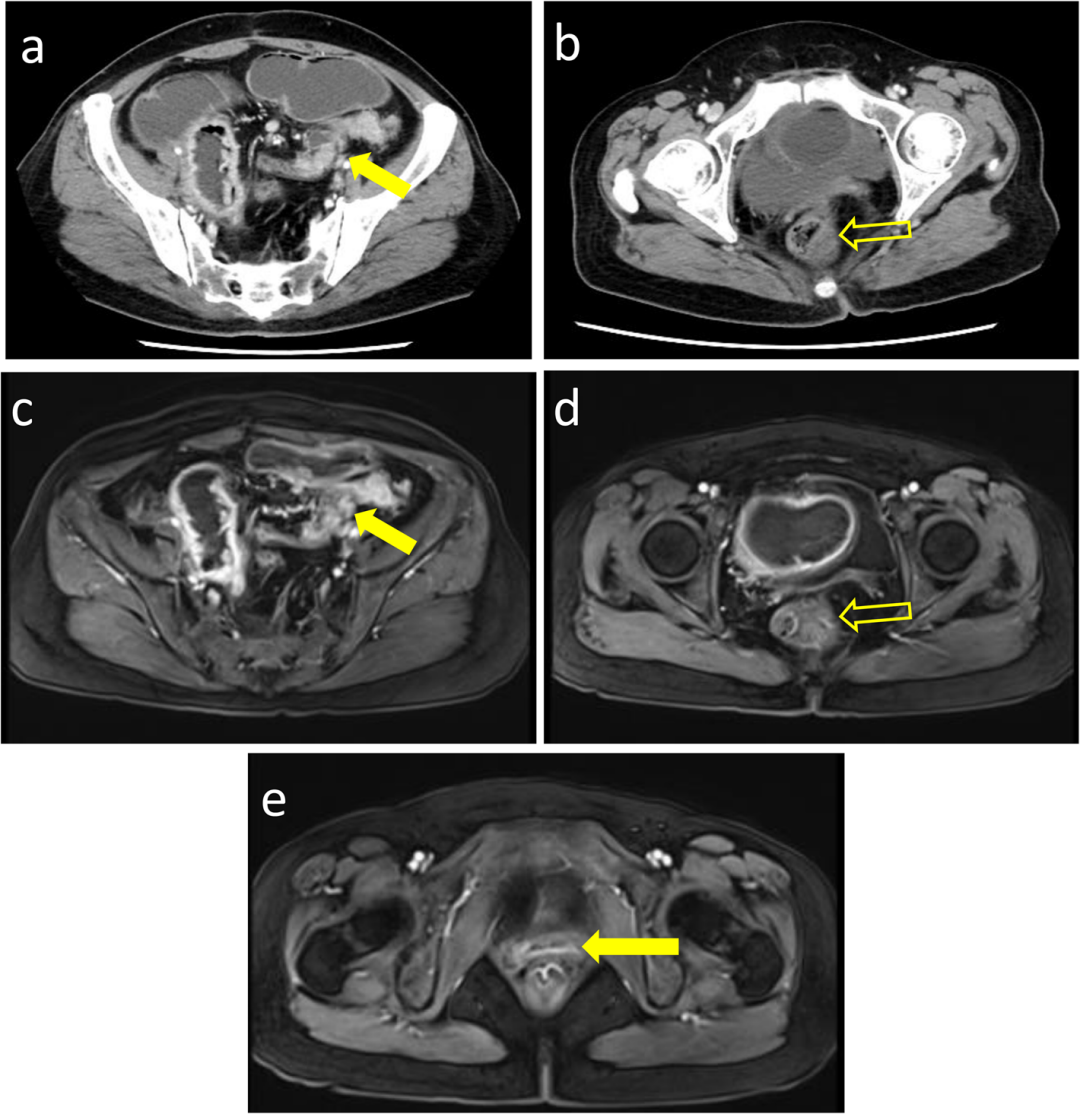
Abdominal computed tomography (CT) scan. CT scan revealed lower intestinal obstruction with a thickened bowel wall in the terminal ileum (*solid yellow arrow* in (**a**) and upper sigmoid colon (*feint yellow arrow* in (**b**). Abdominal magnetic resonance (MR) revealed a thickened bowel wall in the terminal ileum (*solid yellow arrow* in (**c**) and upper sigmoid colon (*feint yellow arrow* in **d**). MR imaging also showed an unremarkable uterus stump (*solid yellow arrow* in **e**)

**Fig. 2:**
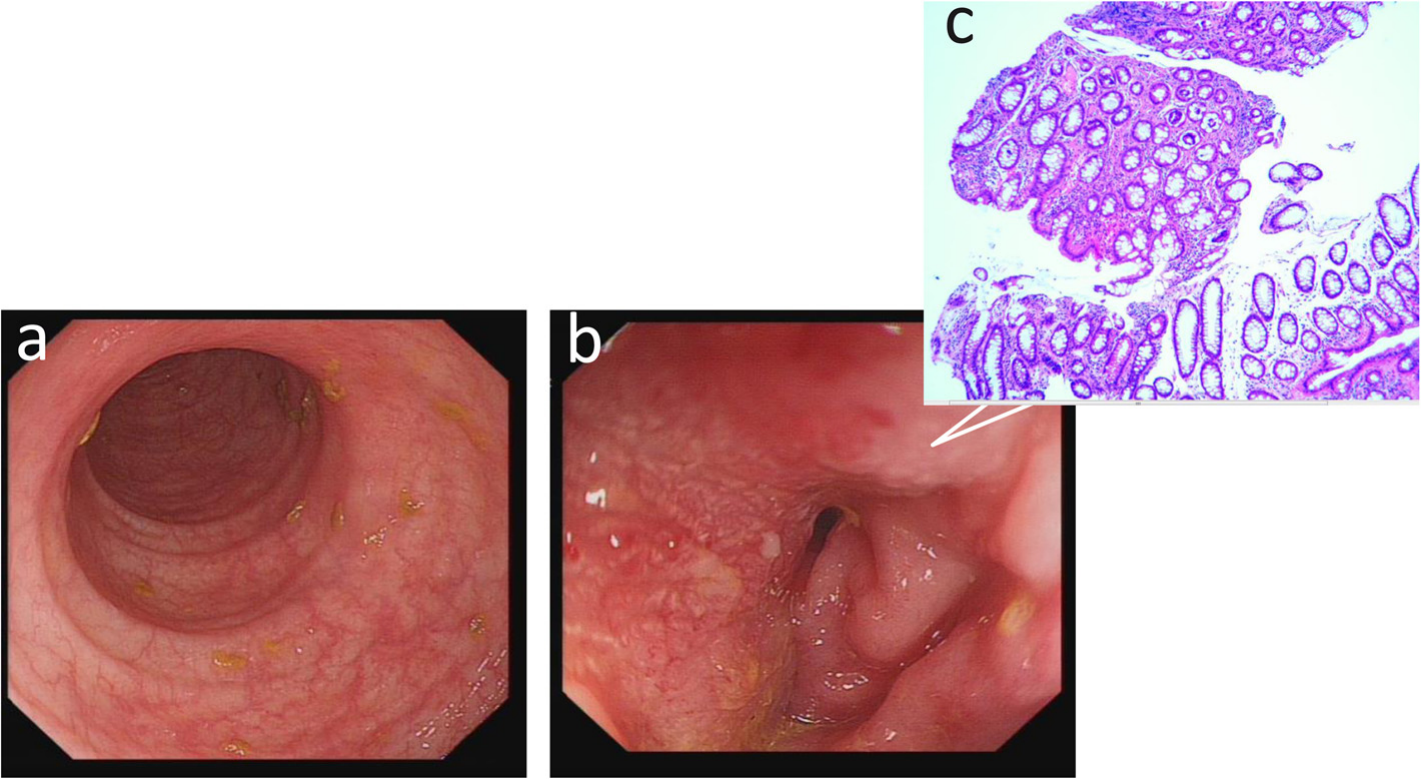
Colonoscopy and biopsy. Colonoscopy confirmed that the intestinal mucosa was smooth without ulcers or lumps **(a)** except sigmoid colon luminal narrowing **(b)**. Pathology result of the narrowing site biopsy showed mucosal chronic inflammation **(c)**

On physical examination, her vital signs were stable. There were no lesions in the oropharynx and nasopharynx. She had abdominal light distension and a loud gurgling sound. No abdominal tenderness and rebound tenderness were found. Rectal examination and bimanual vaginal examination had no obvious abnormalities including lump and bleeding. Analysis of blood tests was unremarkable except the elevating of squamous cell carcinoma antigen (SCCA) and CA125. An erect abdominal plain radiograph was rechecked, showing the upper left intestine expanding and fluid levels with a stepladder pattern. Abdominal contrasted computed tomography (CT) showed intestinal obstruction with a thickened bowel wall in the terminal ileum and upper sigmoid colon (Fig. 1a, b). Abdominal magnetic resonance imaging (MRI) indicated the same result (Fig. 1c, d) and an unremarkable uterus stump (Fig. 1e). Colonoscopy showed a narrowed sigmoid colon lumen with smooth mucosa, and the pathological results indicated mucosal chronic inflammation (Fig. 2). Chest CT and X-ray had no obvious abnormity.

**Fig. 3:**
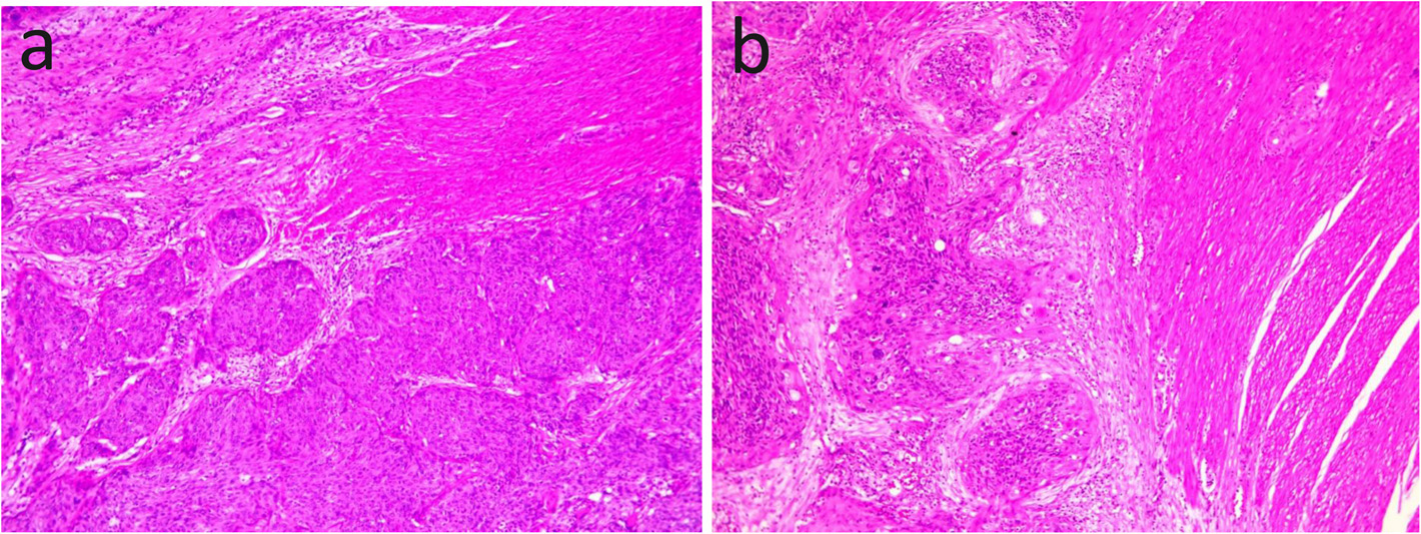
Postoperative pathological examination. Microscopic examination of the intestinal tumor demonstrated a full-thickness wall of sigmoid colon and serosa, and the muscular layer of the terminal ileum had metastatic squamous cell carcinoma. Cancer embolus was seen in the vessel, and the peri-intestine lymph node also had metastatic squamous cell carcinoma. **a** small intestine and **b** sigmoid colon (staining with hematoxylin and eosin, ×100 magnification)

The patient was fasted and received passive gastric decompression along with total parenteral nutrition on admission. Abdominal distention eased on the first day. In consideration of the patient’s condition and the imaging examination and colonoscopy results, exploratory laparotomy was performed. Widespread intestinal adhesion and two firm tumors (2*2 cm and 3*2 cm separately) with a local thickened intestine wall at the end of the ileum and upper sigmoid colon were noted. She underwent segmental intestine resection and had end-to-end anastomosis. The postoperative pathological diagnosis showed squamous cell carcinoma (Fig. 3). After the operation, up until now (4 months), the patient has been treated with four cycles of a docetaxel-cisplatin combination chemotherapy regimen (day 1, 75 mg/m^2^ docetaxel; days 1–3, 25 mg/m^2^ cisplatin, per 21 days).

### Discussion

In the present case, the patient underwent segmental intestine resection, and the pathological result indicated squamous cell carcinoma. Excluding possible primary lesions like oropharynx, nasopharynx, lung, and esophagus cancer, with the characteristics of outside-to-inside invasion from the pathology result, indicating the primary lesion in the abdominal or pelvic cavity, cervical cancer with small intestine and sigmoid colon metastases was diagnosed.

After precise calculation, the 10-year incidence of distant metastases was 3 % for stage IA, 16 % for stage IB, 31 % for stage IIA, 26 % for stage IIB, 39 % for stage III, and 75 % for stage IVA in cervical cancer patients [7]. The most frequently observed metastatic sites were the lungs, bones, liver, supraclavicular nodes, and para-aortic nodes etc. (Table 1). Unusual metastases can be seen in the skin and soft tissues [8], breast [9], pericardium [10], umbilical region [11], labia and introitus area [12], thyroid gland [13], oral cavity [14], and skeletal muscle [15]. Cervical cancer metastatic to the intestine is a rare occurrence. Table 2 provides a comprehensive review of the published cases of intestine metastases from cervical cancer in the English literature dating back to 1976. Things did not go the way we thought because not only do cervical cancer metastases to the intestine occur at an advanced tumor stage, but they also occur at an early stage, even at stage IA. The case presented here is the first report of cervical cancer with simultaneous small intestine and sigmoid colon metastases.

**Table 1:** The main metastatic sites of cervical cancer

metastatic sites^a^ [26]	Percent
Nodes	8.6
Supraclavicular	3.0
Para-aortic	3.0
Inguinal	2.3
Mediastinal	1.7
Iliac	1.3
Cervical	0.8
Axillary	0.5
Other	1.8
Lung	5.7
Bone	3.8
Peritoneum	0.6
Liver	2.2
Gastrointestinal tract	8.0
Stomach [17]	<2.0
Ileum [27]	1.2–3.2
Spleen [28]	1.6–30.0
Ovary [29]	1.3–6.3
Heart [30]	1.2
Brain [31]	0.4–1.2
Skin and subcutaneous tissue [32]	0.1–2.0

^a^Modified and updated from [26]

**Table 2:** Intestine metastases from cervical cancer as reported in the literature

No.	Author [Ref.]	Year	Age	Pathologic type	Stage of SCCA of cervix at diagnosis	Previous treatment	Interval time	Symptom	Metastasis sites	Confirmation of diagnosis	Treatment	Outcome
1	Bradley Watson [27]	1976	47	Adenosquamous carcinoma	Stage IV	None	Synchronous	Intermittent central abdominal pain associated with vomiting	Small intestine	Laparotomy	Segmentary intestinal resection	NA
2	Gurian, L. [17]	1981	64	Squamous cell carcinoma	Stage IIIB	None	Synchronous	Occult bleeding	Duodenum	Endoscopy	Refused surgical intervention	Death
3	Mathur, S. K. [18]	1984	35	Squamous cell carcinoma	Stage IV	None	Synchronous	Central abdominal angina, persistent vomiting and constipation	Terminal ileum	Laparotomy	Right hemicolectomy	Recovery
4	Christopherson, W. [33]	1985	42	Squamous cell carcinoma	Stage IIIB	NA	2 years	Intermittent nausea and vomiting, upper abdominal pain	Ileum, transverse colon	Laparotomy	Segmentary intestinal resection	Recovery
5	Hulecki, S. J. [34]	1985	48	Squamous cell carcinoma	Stage IB	NA	7 years	Gross hematuria from the conduit	Ileum	Endoscopy	Laparotomy	Recovery
6	Misonou, J. [16]	1988	69	Squamous cell carcinoma	Stage IA	Hysterectomy	13 years	Sudden onset of pan-peritonitis	Small intestine	NA	NA	NA
7	Singla, M. [35]	2011	48	Squamous cell carcinoma	NA	Radiation therapy	2 years	Right hypochondrium pain	Hepatic flexure of colon	Laparotomy	Right extended hemicolectomy	Recovery for 2 years
8	Kanthan, R. [19]	2011	49	Squamous cell carcinoma	Stage IIA	Chemotherapy and radiation treatment	2 years	Upper-gastrointestinal bleeding	Duodenum	esophagogastroduodenoscopy	None	Died of multiple organ failure
9	Lee T.H. [36]	2011	50	Squamous cell carcinoma	Stage IIA	Hysterectomy with systemic chemotherapy	2 years	Epigastric pain	Ampulla of vater	Endoscopy	Chemotherapy	NA
10	Raphael, J. C. [37]	2011	57	Squamous cell carcinoma	Stage IV	None	Synchronous	Persistent epigastric pain and vomiting	Pyloroduodenal region	Endoscopy	chemotherapy	NA
11	Sugimoto, T. [38]	2013	84	Adenocarcinoma	Stage III	Radiation therapy	3 months	Epigastric pain	Ileum	Laparotomy	The necrotic part of the ileum resection	4 months survival
12	Joshi, S. R. [39]	2013	50	Squamous cell carcinoma	Stage II	Wertheim’s hysterectomy	5 months	Abdominal pain, vomiting and intermittent fever	Ileocaecal region	Laparotomy	Segmentary intestinal resection	NA
13	Datta, S. [40]	2013	55	Squamous cell carcinoma	Stage IIB	Chemoradiation	3.5 years	Abdominal pain, vomiting, constipation	Ileocaecal region	Laparotomy	Right hemicolectomy	NA
14	Barlin, J. N. [41]	2013	37	Adenosquamous carcinoma	Stage IB	Radical hysterectomy	1.5 years	Hematochezia	Sigmoid colon	Colonoscopy	Rectosigmoid resection	Recovery
15	Iliescu, L. [42]	2014	70	Squamous cell carcinoma	Stage IIA1	Radiation therapy followed by curative surgery	2 years	Intermittent subocclusive symptoms, fatigue, nausea	Terminal ileum	Laparotomy	Segmentary intestinal resection	NA
16	Debasish, B. [43]	2014	43	Squamous cell carcinoma	NA	Total abdominal hysterectomy	8 months	Symptoms of chronic intestinal obstruction	Terminal ileum	Laparotomy	Right hemicolectomy	NA
17	Nagarekha, K. [44]	2014	50	Squamous cell carcinoma	NA	Hysterectomy with bilateral salpingo-oophorectomy	3 months	Vomiting and abdominal pain	Jejunum	Laparotomy	Segmentary intestinal resection	NA
18	Hui Qiu [45]	2015	46	Squamous cell carcinoma	Stage IIB	Chemoradiotherapy	4 years	Acute abdominal pain	Ileocaecal region	Laparotomy	Segmentary intestinal resection	Recovery for 2 years

*NA* not available from original literature

The intestine metastases usually occur through the lymphatics to the bowel serosa and less commonly via intraperitoneal dissemination, direct spread, and hematogenous spread [16–18]. As for the present case, another explanation is surgical factor, for tumor dissemination may be caused by reckless operation. Although the possible metastatic route is distinct, the rarity of intestine metastases is still unclear now [19]. Sigmoid colon metastases are rarely seen and may be because of the relatively short intestinal segment. While small intestine accounted for a large space in the enterocelia. So, the low incidence rates of small intestine metastases are notable, and several associated mechanisms have been raised as follows [20–24]: (1) the intestine has abundant immune protection with numerous lymphoid cells and large secretions of IgA in the mucosa and submucosa of the intestine; (2) a rapid refresh rate of small intestinal mucosa may inhibit the tumorigenesis; (3) liquefied chyme may cause less mucosal irritation, then reduces mechanical injury and inflammation [19].

In general, a small intestine and sigmoid colon metastatic tumor indicates a poor prognosis. Bleeding and obstruction, as well as non-specific symptoms such as abdominal discomfort, gas distension, and vomiting, are common clinical features. Some reasons may cause a misdiagnosis and delay the treatment: (1) non-specific gastrointestinal symptoms may easily be seen as a symptom of tumor progression or adverse drug reaction; (2) lack of awareness of metastatic tumor; (3) ordinary CT scanners cannot find minimal lesions, especially in the small intestine. Once intestine metastasis is suspected, contrast-enhanced CT, endoscope, and even exploratory laparotomy should be operated. Typical features of intestinal metastases include intestinal wall thickening and stiffness, submucosal spread, and ulcers. Typically, metastases are submucosal or subserosal, which make the primary and secondary tumors easily distinguishable; besides, cytokeratin immunohistochemistry may help to differentiate the two. Metastatic cervical carcinoma is usually positive for CK7, epithelial membrane antigen, and CK5/6 and negative for CK20 [25]. The treatment for a small intestine and sigmoid colon metastatic tumor from cervical squamous cell carcinoma remains debatable because of the lack of enough cases to compare the efficacy of different treatments. Laparotomy seems to be the common choice if the patients are physically capable (Table 2). Chemotherapy could also be employed as a palliative treatment.

## Conclusions

This report presents a rare case of small intestine and sigmoid colon metastases of cervical cancer that caused obstruction. Clinicians should be aware that intestine metastasis must be considered in the differential diagnosis of acute abdomen in patients with cervical cancer even at an early tumor stage.

### Consent

Written informed consent was obtained from the patient for publication of this Case report and any accompanying images. This report adhered to the tenets of the Declaration of Helsinki.

## Abbreviations

CT: computed tomography
FIGO: International Federation of Gynecology and Obstetrics
MRI: magnetic resonance imaging
SCCA: squamous cell carcinoma antigen
